# Downregulation of microRNA‐494 drives mitochondrial biogenesis and function in trained muscle

**DOI:** 10.1113/EP092977

**Published:** 2025-08-25

**Authors:** Natália Pálešová, Klára Gabrišová, Jana Babulicová, Patrik Krumpolec, Zuzana Kovaničová, Tímea Kurdiová, Salvatore Modica, Christian Wolfrum, Jozef Ukropec, Barbara Ukropcová, Miroslav Baláž

**Affiliations:** ^1^ Biomedical Research Center Slovak Academy of Sciences Bratislava Slovakia; ^2^ Institute of Food, Nutrition and Health ETH Zurich Schwerzenbach Switzerland; ^3^ Institute of Pathophysiology, Faculty of Medicine Comenius University Bratislava Slovakia; ^4^ Department of Animal Physiology and Ethology, Faculty of Natural Sciences Comenius University Bratislava Slovakia

**Keywords:** exercise, metabolism, microR‐494, mitochondria, skeletal muscle, type 2 diabetes

## Abstract

MicroRNAs (miRNAs) are key regulators of cellular processes, including mitochondrial function and energy metabolism. This study explores the regulation of miR‐494 in skeletal muscle and circulation, investigating its response to exercise training and an acute exercise bout, its association with metabolic disorders, and the effects of electrical pulse stimulation (EPS). In addition, it validates the gene targets and physiological role of miR‐494 using gain‐ and loss‐of‐function studies in primary human skeletal muscle cells. We demonstrate that miR‐494 levels in both skeletal muscle and circulation are influenced by long‐term exercise training, which induces adaptive changes, but remain unaffected by an acute bout of exercise. EPS does not alter miR‐494 levels in cultured primary human skeletal muscle cells. Moreover, muscle miR‐494 levels remain unchanged under various metabolic challenges, including obesity and type 2 diabetes. Genetic manipulation of miR‐494 in primary human skeletal muscle cells modulates mitochondrial biogenesis and function, as well as lipid metabolism, through targeting *PGC1A* and *SIRT1*. Injection of a miR‐494 inhibitor into skeletal muscle of mice supports the role of miR‐494 in regulating *Pgc1α* mRNA, suggesting potential therapeutic implications. These findings highlight miR‐494 as a significant modulator of mitochondrial dynamics and energy metabolism in skeletal muscle.

## Abstract (highlights)

- **What is the central question of this study?**
  How is miR‐494 regulated in skeletal muscle and circulation and what are its responses to exercise training and an acute exercise bout, its association with metabolic disorders, and the effects of electrical pulse stimulation?
- **What is the main finding and its importance?**
  miR‐494 is downregulated in trained muscle but not affected by acute exercise and the presence of metabolic disease. Inhibition of miR‐494 promotes mitochondrial biogenesis and respiration in primary human skeletal muscle cells through upregulation of PGC1A and SIRT1. These results highlight miR‐494 as a significant modulator of mitochondrial dynamics and energy metabolism.

## 1 INTRODUCTION

Physical activity is a vital component of daily life and provides many health benefits, including reduced risk of metabolic, cardiovascular, oncological and neurodegenerative disorders (Garber et al., 2011; Myers et al., 2019; Stranahan & Mattson, 2012). In contrast, physical inactivity substantially increases the risk of numerous health conditions and ultimately shortens life expectancy. A recommended dose of 150 min of moderate‐intensity physical activity per week can reduce the risk of developing type 2 diabetes by up to 26% (Wahid et al., 2016). Exercise induces adaptive molecular changes in the skeletal muscle, enhancing its capacity for glucose uptake and oxidation, glycogen storage and utilisation (Flockhart et al., 2021; Park et al., 2020), and fatty acid oxidation (Bruce et al., 2006; Jeppesen et al., 2012; Muscella et al., 2020). These benefits are largely mediated by the stimulation of mitochondrial biogenesis (Halling et al., 2017; Yokokawa et al., 2018), increased tissue respiratory capacity (Flockhart et al., 2021), and upregulation of specific enzymatic activities supporting mitochondrial function and recycling, such as mitophagy (He et al., 2020), ultimately leading to improved insulin sensitivity (Bergman & Goodpaster, 2020; Bruce et al., 2006). Exercise also induces significant changes in skeletal muscle at the level of microRNAs (miRNAs) (McLean et al., 2015), which have emerged as central players in the control of various physiological and pathophysiological processes. MiRNAs are regulated by physical activity and contribute to the adaptive response to exercise (Massart et al., 2021; Safdar et al., 2009), thereby mediating some of the beneficial effects of exercise on metabolic health. One such miRNA regulated by muscle contraction is miR‐494, which has been implicated in several physiological processes, including angiogenesis and osteogenesis (Ding et al., 2021; Esser et al., 2017), as well as cell proliferation and apoptosis (He et al., 2017). Dysregulation of miR‐494 has been linked to serious conditions such as hepatocellular carcinoma (Fornari et al., 2015), breast cancer (Zhan et al., 2018) and gestational diabetes mellitus (He et al., 2017). In skeletal muscle, the expression of miR‐494 influences both muscle metabolism and functional capacity; its overexpression reduces the formation of fast oxidative, force‐generating myofibres (Iwasaki et al., 2021). A naturally occurring reduction in miR‐494 levels during muscle differentiation is associated with increased expression of mitochondrial transcription factor A (TFAM) and Forkhead box j3 (FOXJ3), which promote mitochondrial biogenesis and enhance muscle adaptation to exercise (Yamamoto et al., 2012). Additionally, miR‐494 levels in muscle decrease following 8 weeks of voluntary wheel running (Sun et al., 2016), highlighting miR‐494 as an interesting candidate with the capacity to modulate skeletal muscle energy metabolism. In the present study, we investigated the regulation of skeletal muscle and circulating miR‐494 in response to supervised exercise training and its potential as a marker of metabolic disease progression along the spectrum from obesity to prediabetes and type 2 diabetes. The role of miR‐494 in regulating mitochondrial function and metabolic activity was examined using gain‐ and loss‐of‐function studies in primary human skeletal muscle cells. We provide clear evidence that miR‐494 is downregulated in trained muscle and plays an important role in the control of mitochondrial biogenesis and activity through modulation of *PGC1A*.

## 2 METHODS

### 2.1 Ethical approval

Our research complies with the journal's policies regarding human and animal experimentation. All clinical studies and procedures were approved by the Ethics Committee of the University Hospital Bratislava, Comenius University Bratislava (approval date: 1 July 2008) and/or the Ethics Committee of the Bratislava Region (approval date: 5 March 2011), and were conducted in accordance with the standards of the *Declaration of Helsinki* 2013. The exercise intervention study (Cohort 1) was registered in the UK's Clinical Study Registry under ISRCTN95105191. All participants provided witnessed written informed consent prior entering the study. The mouse study was carried out according to the ETH Zurich Policy on Experimental Animal Research, followed the ethical framework and guidelines laid down by the Animal Welfare Committee of ETH Zurich and was approved by the Veterinary office of the Canton of Zurich (registration number ZH183/2011). We fully adhere to the ethical principles upheld by the journal and have complied with the animal ethics checklist. All necessary steps were taken to minimise animal pain and suffering. Further details on the mouse study are provided in the section ‘Mouse study’.

### 2.2 Study design

#### 2.2.1 Exercise intervention study (Cohort 1)

**FIGURE 1:**
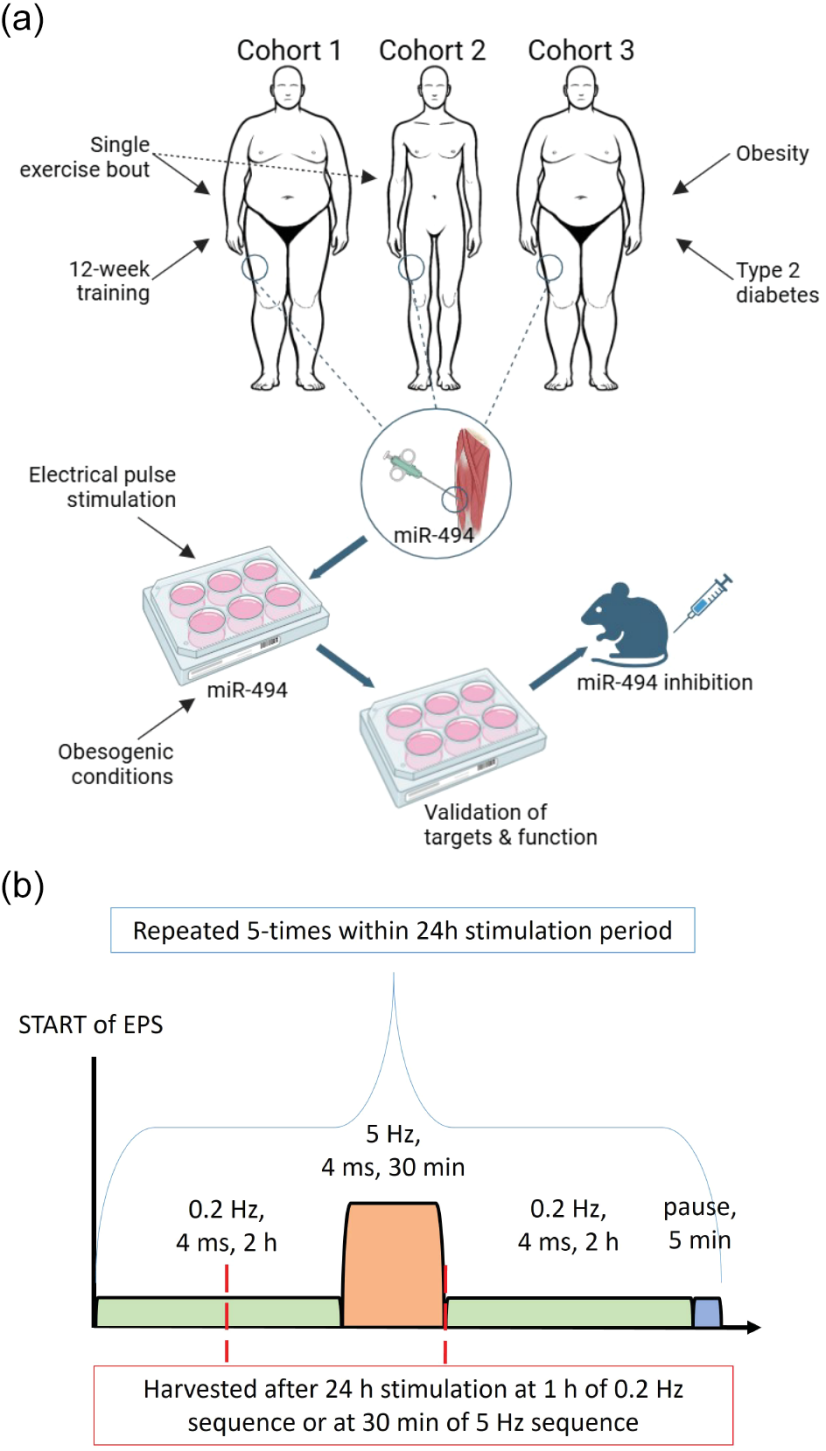
Schematic overview of the experimental design and intermittent electrical pulse stimulation protocol. (a) in vivo and in vitro experimental approaches. In Cohort 1, we examined effects of exercise training, and an acute exercise bout performed before and after 12 weeks’ training in individuals with pre‐obesity and obesity. Cohort 2 allowed access to the muscle biopsies from young trained individuals taken at baseline pre‐exercise state and immediately post 1‐h aerobic exercise (stationary cycle), and Cohort 3 was designed to examine effects of obesity, pre‐diabetes and newly diagnosed type 2 diabetes on muscle energy metabolism. Regulation of miR‐494 by electrical pulse stimulation and an obesogenic environment (saturated fatty acid palmitate) and functional gain‐ and loss‐of‐function studies were performed on primary human skeletal muscle cells in vitro, followed by a proof‐of‐concept experiment in mice in vivo. (b) Schematic overview of the intermittent EPS protocol, in which 2‐h subthreshold frequency stimulations (0.2 Hz, 4 ms) were combined with 30‐min periods of higher frequency stimulation and 5‐min rest periods.

Fourteen sedentary individuals with pre‐obesity or obesity (7 males and 7 females; age 36.1 ± 1.3 years; BMI 31.6 ± 0.7 kg m^−2^) with at least two attributes of metabolic syndrome underwent a 12‐week supervised training programme (Figure 1a). Supervised 60‐min training sessions were conducted three times per week. The training protocol and overall study design were previously described (Kurdiova et al., 2014). In addition, we examined the effects of a single 60‐min bout of indoor cycling performed at 75% of heart rate maximum. The same relative intensity was applied pre‐ and post‐training, with adjustments made to account for training‐induced changes in cardiopulmonary performance (Table 1). Skeletal muscle biopsies and blood samples were collected 48 h after the final training session to avoid acute exercise effects and to specifically assess training‐induced adaptations. The fitness outcomes of this exercise intervention (${\dot{V}}_{O_{2}\max}$ and visceral adiposity) were previously published (Kurdiova et al., 2014). In the original study, Cohort 1 included 16 individuals with pre‐obesity or obesity. For the current analysis, two individuals were excluded as we did not have sufficient skeletal muscle sample for miRNA extraction and miR‐494 quantification.

**TABLE 1:** Clinical characteristics of the study Cohort 1 and effects of 12 weeks’ training.

	Pre‐training (*n* = 14)	Post‐training (*n* = 14)
Age (years)	36.1 ± 5.1	—
Body weight (kg)	95.2 ± 10.4	95.2 ± 10.6
BMI (kg/m^2^)	31.9 ± 2.4	31.6 ± 2.6
Lean body mass (kg)	61.5 ± 10.3	61.4 ± 10.3
Body fat (%)	35.7 ± 7.0	35.2 ± 7.1
Waist circumference (cm)	106.0 ± 7.8	102.5 ± 7.0^**^
Systolic BP (mmHg)	126.4 ± 13.0	122.12 ± 11.4
Diastolic BP (mmHg)	80.4 ± 12.0	80.3 ± 11.0
Resting HR (counts/min)	78.7 ± 9.3	73.5 ± 8.0
Maximal HR (counts/min)	173.9 ± 8.7	174.2 ± 8.6
${\dot{V}}_{O_{2}\max}$ (mL/min/kg LBM)	31.8 ± 9.6	39.2 ± 8.1^*^
Fasting glycemia (mM)	5.21 ± 0.55	5.58 ± 1.37
2‐hour glycemia in oGTT (mM)	7.29 ± 1.75	6.60 ± 1.41
Total cholesterol (mM)	5.01 ± 1.19	4.55 ± 0.93
HDL cholesterol (mM)	1.36 ± 0.36	1.23 ± 0.30
Triglycerides (mM)	1.74 ± 1.02	1.58 ± 0.69
Fasting insulin (mU/l)	8.89 ± 4.47	8.23 ± 5.38
HOMA index of IR	2.08 ± 1.09	2.03 ± 1.25

*Note*: Data are expressed as means ± SD; differences between the groups were evaluated using paired *t*‐test, and significant differences are marked in bold and indicated as *P < 0.05 and **P < 0.01. ${\dot{V}}_{O_{2}\max}$ data were previously published in Kurdiova et al. (2014). BP, blood pressure; HR, heart rate; LBM, lean body mass; oGTT, oral glucose tolerance test.

#### 2.2.2 Acute exercise cohort (Cohort 2)

Healthy, physically active young males (*n* = 6, age 24.5 ± 2.7 years, BMI 25.6 ± 4.6 kg/m^2^) completed a 60‐min session of high‐intensity endurance exercise (stationary cycling) at 75–80% of their maximum heart rate (Figure 1a). Vastus lateralis muscle biopsies were obtained using the Bergström needle technique in the morning, both before the acute exercise bout and immediately after its completion.

#### 2.2.3 Sedentary men with normal weight, pre‐obesity or obesity, prediabetes and type 2 diabetes (Cohort 3)

Forty‐eight sedentary, middle‐aged men were classified into four groups: healthy lean individuals (*n* = 12), individuals with pre‐obesity or obesity (*n* = 12; 7 with overweight, 5 with obesity), and patients with newly diagnosed prediabetes (*n* = 12) or type 2 diabetes (*n* = 12; Figure 1a). The obesity level in the last three groups was comparable. Individuals with any diagnosed chronic disease or regular use of pharmacotherapy, including lipid and glucose‐lowering therapy, were not eligible to enter the study. Clinical characteristics of Cohort 3 have been previously published (Kurdiova et al., 2014). In the original study, Cohort 3 consisted of 99 individuals. For the current analysis, we randomly selected 12 individuals per group (*n* = 48 in total), based on the availability of skeletal muscle samples for miRNA extraction and miR‐494 quantification. Presence of impaired fasting glucose (5.6–6.9 mmol/L) and/or impaired glucose tolerance assessed by the 2‐h oral glucose tolerance test (7.8–11.0 mmol/L) (Kurdiova et al., 2014) was used to diagnose prediabetes. The oral glucose tolerance test was also used to diagnose type 2 diabetes. All individuals in the T2D group were newly diagnosed and had not yet commenced any glucose‐lowering medication at the time of examination. The characteristics of the cohort are summarised in Table 2.

**TABLE 2:** Clinical characteristics of study Cohort 3.

	Lean (*n* = 12)	Pre‐obesity/obesity (*n* = 12)	Pre‐diabetes (*n* = 12)	T2D (*n* = 12)
Age (years)	44.3 ± 8.6	42.6 ± 5.9	43.8 ± 10.3	49.2 ± 8.5
BMI (kg/m^2^)	23.9 ± 1.8	29.6 ± 2.0^***^	29.6 ± 3.7^***^	31.6 ± 3.8^***^
Waist circumference (cm)	89.2 ± 9.3	104.6 ± 8.2^***^	106.7 ± 11.5^***^	110.0 ± 11.4^***^
Body fat (%)	18.8 ± 3.3	27.9 ± 5.3^***^	29.0 ± 6.6^***^	31.3 ± 4.1^***^
Adipocyte diameter (µm)	98.3 ± 15.7	120.0 ± 12.4^**^	114.9 ± 17.3^*^	117.0 ± 15.7^**^
Fasting glycaemia (mM)	4.7 ± 0.4	4.9 ± 0.3	5.8 ± 0.7^***^	9.0 ± 2.9^***^
2‐hour glycemia in oGTT (mM)	5.9 ± 1.1	5.20 ± 1.8	8.8 ± 2.0^**^	13.2 ± 3.5^**^
M‐value/insulin	0.14 ± 0.05	0.09 ± 0.04^*^	0.08 ± 0.04^**^	0.05 ± 0.04^***^
Triglycerides (mM)	1.19 ± 0.67	1.65 ± 0.97	1.71 ± 1.07	1.97 ± 1.03
Total cholesterol (mM)	4.44 ± 0.93	4.86 ± 0.83	5.18 ± 1.58	5.05 ± 0.73
HDL cholesterol (mM)	1.40 ± 0.19	1.26 ± 0.21	1.35 ± 0.24	1.18 ± 0.13
LDL cholesterol (mM)	2.50 ± 0.65	2.79 ± 0.73	3.05 ± 1.28	2.98 ± 0.63
Fasting FFA (mM)	0.63 ± 0.16	0.63 ± 0.19	0.72 ± 0.22	0.67 ± 0.27
FFA during EHC (mM)	0.11 ± 0.04	0.14 ± 0.04	0.14 ± 0.07	0.17 ± 0.10

*Note*: Data are expressed as means ± SD; differences between the groups were analysed using one‐way ANOVA with Bonferroni *post hoc* test; significant differences from lean (control) population (*P* ˂ 0.05) are marked in bold and indicated as ^*^ *P *< 0.05, ^**^ *P *< 0.01 and ^***^ *P *< 0.001. EHC, euglycemic hyperinsulinemic clamp; FFA, free fatty acids; oGTT, oral glucose tolerance test.

### 2.3 Skeletal muscle biopsy

Samples of m. vastus lateralis were taken by percutaneous Bergström needle biopsy technique under local anaesthesia and aseptic conditions as previously described (Kurdiova et al., 2014). In the exercise intervention study (Cohort 1), the sampling was performed in the morning after an overnight fast, both before and 48 h after completing the 12‐week exercise training programme (i.e., after the final training session). Seven participants agreed to undergo an additional biopsy related to acute exercise. In these individuals, the baseline biopsy was followed by an acute exercise bout (a 60‐min training session designed to reach and sustain more than 75% of their actual maximal capacity for at least 45 min), with a second biopsy taken 60–120 min post‐exercise. These acute exercise‐related biopsies were performed both pre‐ and post‐training to assess the adaptive response to an acute exercise bout. In young recreational athletes (Cohort 2), muscle samples were collected before and within 15 min after completing a 60‐min bout of high‐intensity cycling exercise (>75% heart rate reserve) on a stationary cycle. In sedentary men (Cohort 3), muscle biopsies were taken in the morning following an overnight fast. Muscle samples were briefly rinsed in ice‐cold saline, blotted to remove excess fluid, and immediately snap‐frozen in liquid nitrogen. A small portion of each muscle sample (∼80 mg) was used to establish primary human skeletal muscle cell cultures.

### 2.4 Blood collection

Blood was collected into an S‐Monovette venous blood collection tube (Sarstedt, Nümbrecht, Germany) 30 min after cannula insertion into the antecubital vein (SURFLO‐W i.v. Catheter 26 G). Serum was obtained by centrifugation at 1600 *g* for 20 min and 4°C using an Eppendorf 5804R (SW bucket rotor) after 30 min incubation at laboratory temperature.

### 2.5 Mouse study

All animal procedures were approved by the Veterinary Office of the Canton of Zürich. The sample size (*n* = 6) was determined based on previous experiments and comparable studies reported in the literature. Mice were housed under standard conditions (22°C, 12‐h reversed light–dark cycle, dark phase starting at 07.00 h), with ad libitum access to chow (18% proteins, 4.5 % fibres, 4.5% fat, 6.3% ashes, Kliba Nafag, Kaiseraugst, Switzerland) and water. Tibialis anterior muscles of six 12‐week‐old male C57Bl/6 mice (Charles River, Freiburg, Germany) were injected with 5 nmol of miR‐494 inhibitor in the right leg, while the left leg received 20 µL of saline as a negative control. Injections were performed under general anaesthesia (inhalation of isoflurane – 4% for induction and 2% for maintenance). Mice were humanely euthanised in a CO_2_ atmosphere, and the tibialis anterior muscles were harvested 96 h after injection, snap‐frozen in liquid nitrogen, and used for total RNA extraction and gene expression analysis.

### 2.6 Cell culture

Primary human skeletal muscle cells derived from the vastus lateralis muscle were obtained from lean‐to‐overweight healthy donors and cultured as previously described in Kurdiova et al. (2014). In brief, myoblasts (passage 2) were seeded onto collagen‐coated plates at density of 3000 cells/cm^2^. After reaching 80% confluence, differentiation was induced by switching to α‐Minimum Essential Medium (α‐MEM) supplemented with 2% fetal bovine serum (FBS), 0.5% fetuin and 2% penicillin/streptomycin. After 48 h of differentiation, myotubes were transfected with either 30 nM miR‐494 mimic and negative control 1 (Thermo Fisher Scientific, Waltham, MA, USA) or with 60 nM miR‐494 Locked Nucleic Acid (LNA) inhibitor and negative control A (Exiqon, Vedbaek, Denmark) in FBS‐free medium containing antibiotics and Lipofectamine RNAiMAX (Thermo Fisher Scientific) according to manufacturer's instructions. After a 24‐h incubation, the transfection mix was replaced with differentiation medium. Fully differentiated myotubes were harvested 48 h later (i.e., 5 days post‐induction) for RNA isolation and analysis (12‐well plates, non‐starved cells) and for protein extraction (6‐well plates, serum‐starved cells). The cell‐conditioned media (from both RNA and protein plates) were collected and analysed. Cells cultured on 96‐well black plates with transparent bottom were fixed in 4% formaldehyde 6 days after induction of differentiation (72 h after transfection). Lipid droplets, nuclei and cytoplasm were stained with Bodipy, Hoechst, and Syto60 dyes, respectively, and visualised and quantified using the Operetta imaging system (Perkin Elmer, Schwerzenbach, Switzerland). Mitochondrial respiration was analysed using the Extracellular flux analyser XFe96 (Agilent Technologies, Santa Clara, CA, USA). Mitochondrial content was assessed using MitoTracker green staining by immunofluorescence, as well as by quantifying the mitochondrial‐to‐genomic DNA ratio by qPCR. Images of stained cells were taken by the Operetta imaging system and evaluated by software Harmony 2.0 (Perkin Elmer). To study the effect of obesogenic environment on miR‐494 regulation, differentiated primary human skeletal muscle cells were exposed to 80 µM of the saturated fatty acid palmitate coupled to BSA at a 5:1 molar ratio during the final 3 days of the differentiation (Kurdiova et al., 2014). Control cells were exposed to BSA. The original goal of this experiment was to examine the effect of saturated fatty acids on the expression of miR‐494 in differentiated primary human myotubes derived from donors with different metabolic phenotypes: (i) lean and healthy (*n* = 5), (ii) healthy with obesity (*n* = 4), (iii) individuals with obesity and prediabetes (*n* = 5), and (iv) newly diagnosed patients with type 2 diabetes (*n* = 5). As the miR‐494 response to palmitate was found to be independent of donor phenotype, data from all donor groups were pooled and are presented in a single group (*n* = 19).

### 2.7 Electrical pulse stimulation

Electrical pulse stimulation (EPS) serves as an in vitro model of exercise, in which motor neuron activation of muscle fibres is replaced by external electrical stimulation, resulting in repeated contractions of myotubes (Nikolić & Aas, 2019). Fully differentiated myotubes (after 5 days of differentiation), derived from lean‐to‐overweight metabolically healthy men (BMI 24.7 ± 0.6 kg/m^2^, BW 84.4 ± 5.4 kg, age 35.8 ± 7.4 years, *n* = 4), were electrically stimulated using a C‐Pace EP Cell culture stimulator, with C‐dish containing carbon electrodes (Ionoptix, Milton, MA, USA) using two different EPS protocols: (i) continuous stimulation for 24 h, mimicking prolonged low‐intensity exercise (frequency 1 Hz, pulse duration 2 ms), and (ii) intermittent stimulation for 24 h, combining 2‐h subthreshold frequency stimulations (0.2 Hz, 4 ms) with 30‐min periods of higher‐frequency stimulation and 5‐min rest intervals, as illustrated in Table 3 and Figure 1b. The differentiation medium was replaced directly before stimulation. For both protocols, control myotubes were treated identically to EPS stimulated cells by inserting the C‐dish carbon electrode but without connecting it to the pulse generator. After 24 h of stimulation, both cells and cell‐conditioned media were collected (after continuous and after intermittent stimulation—30 min at 5 Hz and 60 min at 0.2 Hz) for RNA extraction, and expression of miR‐494 and its target genes was analysed.

**TABLE 3:** Specification of EPS protocols.

EPS protocol	Sequence	Frequency	Sequence duration	Pulse duration	Duration of the protocol
Continuous stimulation	Ø	1 Hz	24 h	2 ms	24 h
Intermittent stimulation (sequence 1–4 repeated 5 times)	1	0.2 Hz	2 h	4 ms	24 h
	2	5 Hz	0.5 h		
	3	0.2 Hz	2 h		
	4	0 Hz	5 min		

### 2.8 Mitochondrial respiration

Skeletal muscle cells were seeded, differentiated into myotubes, and transfected with miR‐494 mimics, inhibitor and respective controls directly on collagen‐coated 96‐well plates (XFe96 cell culture microplates, Agilent). For measurement of oxygen consumption rate (OCR), the following reagents were diluted in XF medium containing 5 mM d‐glucose (Sigma‐Aldrich, St Louis, MO, USA), 2 mM l‐glutamine (Thermo Fisher Scientific) and 1 mM sodium pyruvate (Thermo Fisher Scientific). The medium was adjusted to pH 7.4, and reagents were injected into the plate at the indicated time points to achieve the following final concentrations: 1 µg/mL oligomycin (ATP‐synthase inhibitor; AdipoGen, Füllinsdorf, Switzerland), 1 µM carbonyl cyanide‐*P*‐trifluoromethoxyphenylhydrazone (FCCP; chemical uncoupler; Sigma‐Aldrich), 3 µM rotenone, and 2 µg/mL antimycin A (electron transport chain inhibitors; Sigma‐Aldrich). Non‐mitochondrial respiration (OCR values following injection of rotenone and antimycin A) was subtracted from overall basal (OCR values prior to injection of oligomycin), uncoupled (OCR values following injection of oligomycin), and maximal respiration (OCR values following injection of FCCP) to calculate basal, uncoupled and maximal mitochondrial respiration rates, respectively. Oxygen consumption data were normalised to protein content per well.

### 2.9 Western blot analysis

After complete removal of the medium, cells were washed in ice‐cold phosphate‐buffered saline and lysed in RIPA buffer supplemented with protease inhibitors (Complete, Roche, Basel, Switzerland) and aprotinin (Sigma‐Aldrich). Concentration of proteins was determined using the BCA assay (Thermo Fisher Scientific). Equal amounts of proteins (20 µg) were separated by PAGE, transferred to an Immobilon‐FL membrane (Millipore, Billerica, MA, USA) and incubated with primary antibodies against peroxisome proliferator‐activated receptor γ coactivator 1‐α (PGC1A; 1:1000; MA5‐15900, Thermo Fisher Scientific, RRID:AB_11152682), SIRT1 (1:1000; MA5‐15677, Thermo Fisher Scientific, RRID:AB_10978176) and AMP‐activated protein kinase α (AMPKα; 1:1000; no. 2793, Cell Signaling Technology, Danvers, MA, USA, RRID:AB_915794). An Odyssey Infrared Imaging System (Li‐Cor Biosciences, Lincoln, NE, USA) was used for signal detection and quantification. Target protein levels were normalised to α‐tubulin (1:5000; no. 926‐42213, Li‐Cor, RRID:AB_2637092), which was stably expressed.

### 2.10 RNA isolation and real‐time PCR

Total RNA was extracted from myotubes using Trizol (Thermo Fisher Scientific) according to the manufacturer's instructions. DNase‐treated RNA (New England BioLabs, Ipswich, MA, USA) was reverse transcribed, and the expression of target genes was quantified using SYBR green and gene‐specific primer pairs on ViiA 7 Real‐Time PCR System (Thermo Fisher Scientific). Ribosomal protein L13A (RPL13A), which was stably expressed in cultured myotubes, served as an internal reference gene. A calibration curve was employed, and PCR efficiency was optimised for each set of primers. All primers used in the study are listed in Table 4.

**TABLE 4:** Sequence of primers used in the study.

*Pgc1a*	FWD	CCCTGCCATTGTTAAGACC
	REV	TGCTGCTGTTCCTGTTTTC
*Sirt1*	FWD	GATGACGATGACAGAACGTCACA
	REV	GGATCGGTGCCAATCATGAG
*Sirt3*	FWD	ACAGCTACATGCACGGTCTG
	REV	TGCTCCCCAAAGAACACAAT
*Rpl13a*	FWD	CCCTCCACCCTATGACAAGA
	REV	GCCCCAGGTAAGCAAACTT
*PGC1A*	FWD	TGAGAGGGCCAAGCAAAG
	REV	ATAAATCACACGGCGCTCTT
*SIRT1*	FWD	AAATGCTGGCCTAATAGAGTGG
	REV	TGGCAAAAACAGATACTGATTACC
*SIRT3*	FWD	TCCCAGTTTCTTCTTTTCGAGT
	REV	GGAAAGCTTCCCCTTGTCAC
*FOXJ3*	FWD	AAAGTGCCTCGATCTAAGGATG
	REV	TCTTCCTTCGGATTGGTGTC
*RPL13A*	FWD	GGACCGTGCGAGGTATGCT
	REV	ATGCCGTCAAACACCTTGAGA
*mtND1*	FWD	CCCTAAAACCCGCCACATCT
	REV	GAGCGATGGTGAGAGCTAAGGT
*gLPL*	FWD	CGAGTCGTCTTTCTCCTGATGAT
	REV	TTCTGGATTCCAATGCTTCGA
*FAS*	FWD	GCAAATTCGAACTTTCTCAGAAC
	REV	GGACCCCGTGGAATGTCA
*DGAT1*	FWD	ACTGGTGGAACTCCGAGTCTGT
	REV	CGAAGCATGGGCTTGTAGAAG

### 2.11 MiRNA quantification and isolation

Total RNA was extracted from skeletal muscle tissue (∼20 mg) and serum (250 µL) using Trizol (Thermo Fisher Scientific) and purified with the miRNeasy mini kit (Qiagen, Germantown, MD, USA) according to the manufacturer's instructions. The presence of miRNA in the samples was verified with the aid of 2100 Bioanalyzer (Small RNA kit, Agilent Technologies). Total RNA from cultured myotubes was extracted using Qiazol (Qiagen). Total RNA and miRNA from skeletal muscle, cultured muscle cells, and serum were reverse transcribed using the miScript II RT kit (Qiagen). Since the serum miRNA yield was low, cDNA was preamplified using the miScript PreAmp PCR kit (Qiagen). MiR‐494 was quantified by real‐time PCR using the Hs_miR‐494_2 miScript primer assay (Qiagen) and miScript SYBR green PCR kit (Qiagen). Small nuclear RNAs *RNU6* (Hs_RNU6‐2_1), *SNORD48* (Hs_SNORD48_1) and *SNORD44* (Hs_SNORD44_1) were stably expressed and used for normalisation of real‐time PCR data (reagents obtained from Qiagen). MiR‐494 content in cell‐conditioned media was normalised to levels of *UniSp2 RNA spike‐in control* (Qiagen).

### 2.12 Quantification and statistical analyses

Our research complies with the journal's statistics policy. For the in vivo mouse study, littermates were used, and no individuals were excluded from the analysis. Sample sizes were determined based on previous experiments using similar methodologies. All cell culture experiments were performed on primary human muscle cells obtained from lean‐to‐overweight healthy donors. All experimental data are presented as individual data points and represent biological replicates, meaning each experiment was performed using cells derived from different donors. Cardiopulmonary fitness (${\dot{V}}_{O_{2}\max}$) and visceral adiposity data for Cohort 1 have been previously published (Kurdiova et al., 2014). In the original study, Cohort 1 consisted of 16 individuals with pre‐obesity or obesity who underwent a 12‐week exercise training programme. In the current study, two individuals were omitted due to insufficient skeletal muscle samples for miRNA extraction and miR‐494 quantification. Clinical characteristics of Cohort 3 have also been published (Kurdiova et al., 2014). The original cohort comprised 99 individuals. For the current study, we randomly selected 12 patients per group (48 patients in total) based on the availability of skeletal muscle samples suitable for miRNA extraction and miR‐494 quantification. Results are reported as means ± SD. Comparisons between two groups were performed using a two‐tailed paired Student's *t*‐test. For comparison among multiple groups, one‐way ANOVA followed by the Bonferroni *post hoc* test was used. Pearson's correlation coefficient was calculated, and all statistical analyses were performed using GraphPad Prism 7 (GraphPad Software, San Diego, CA, USA). Statistical significance is indicated in tables as ^*^ *P *< 0.05, ^**^ *P *< 0.01 and ^***^ *P *< 0.001.

## 3 RESULTS

### 3.1 A 12‐week exercise training programme improved physical fitness in sedentary individuals with obesity

Fourteen middle‐aged, sedentary individuals with pre‐obesity or obesity (Cohort 1, Figure 1a, characteristics in Table 1), each presenting with at least two components of metabolic syndrome, were metabolically phenotyped before and after completing the 12‐week supervised exercise training programme. The intervention increased maximal aerobic capacity (${\dot{V}}_{O_{2}\max}$) by 23% (*P* = 0.048; Table 1; Kurdiova et al., 2014) and resulted in a 3.3% reduction in waist circumference (*P* = 0.008). The parameters of adiposity (subcutaneous and visceral abdominal fat content), circulating lipids (triglycerides, free fatty acids, cholesterol), prediabetes (fasting and 2‐h glycaemia) and insulin resistance (fasting insulinaemia, HOMA‐IR) remained unchanged (Table 1).

### 3.2 Skeletal muscle and circulating mir‐494 are regulated by a supervised exercise training programme

**FIGURE 2:**
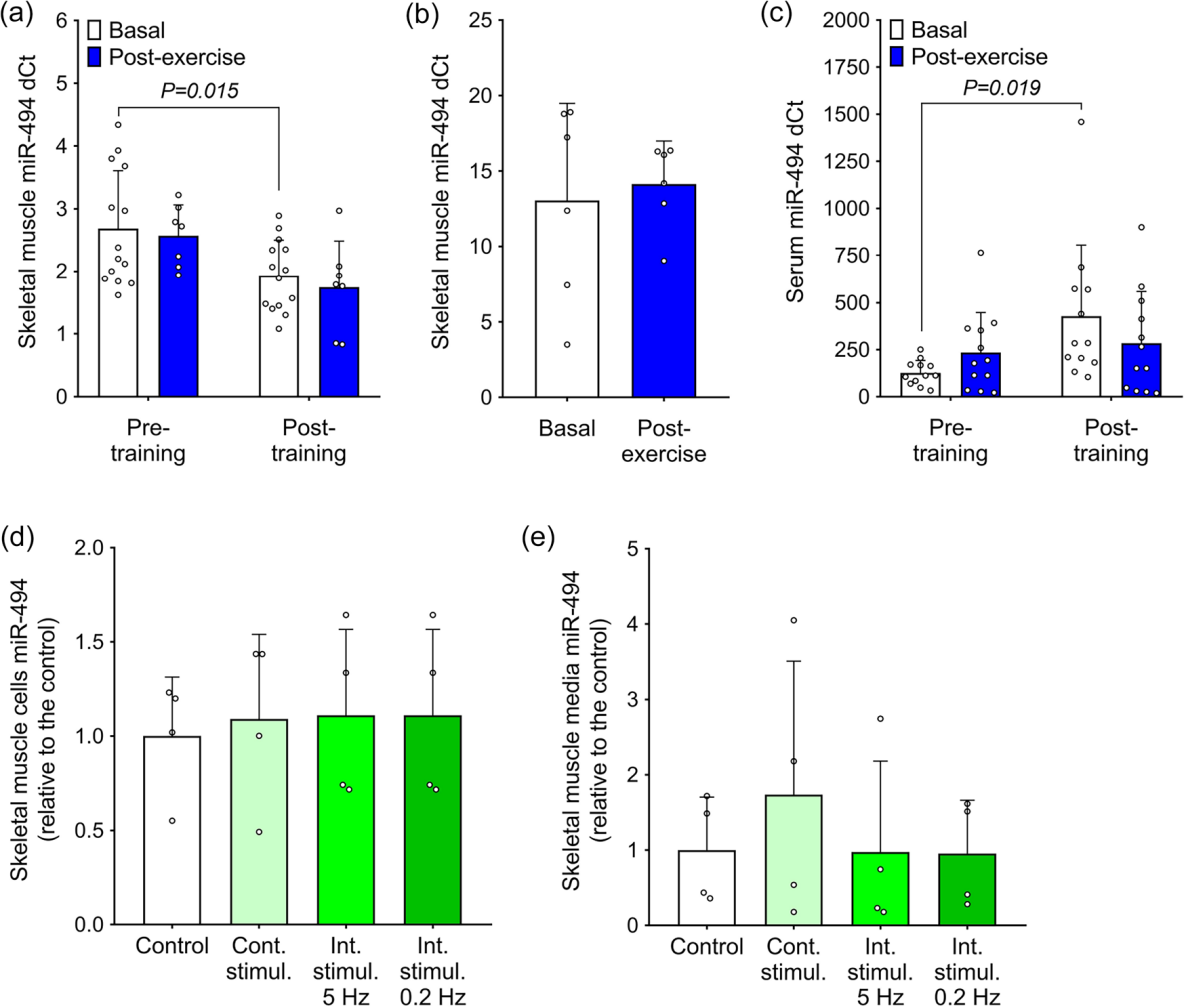
Regulation of miR‐494 in muscle and circulation by exercise in vivo and by exercise mimicking in cultured myotubes in vitro. (a) Effect of a 12‐week exercise training (*n* = 14) and an acute 60‐min exercise bout (*n* = 7) on skeletal muscle miR‐494 levels (Cohort 1, data are normalised to arithmetic mean of *RNU6*, *SNORD44* and *SNORD48*). (b) Effect of an acute 60‐min exercise bout (*n* = 6) on skeletal muscle miR‐494 content in young, trained volunteers (Cohort 2, data are normalised to levels of *RNU6*). (c) Effect of a supervised 12‐week training (*n* = 12) and an acute 60‐min exercise bout (*n* = 12) on serum miR‐494 levels in sedentary individuals with pre‐obesity or obesity (Cohort 1, data are normalised to levels of *RNU6*). (d, e) Effect of continuous (Cont.) and intermittent (Int.) electrical pulse stimulation on miR‐494 levels in primary human skeletal muscle cells derived from overweight‐to‐obese healthy donors (*n* = 4, data are normalised to the expression levels of *SNORD44*) and cell‐conditioned media (*n* = 4; data are normalised to levels of *UniSp2 RNA spike‐in control*).

The 12‐week supervised exercise training programme reduced baseline levels of miR‐494 in resting skeletal muscle (measured 4–5 days after the final exercise session) by 28% (*P* = 0.015; Figure 2a). However, a single 60‐min session of exercise did not affect muscle miR‐494 content in either sedentary or trained individuals (Figure 2a). We next investigated whether the lack of miR‐494 regulation by acute exercise was due to the timing of muscle sampling (60–120 min) after completion of the exercise session. Therefore, we analysed muscle miR‐494 levels in young, trained men (Cohort 2) who underwent muscle biopsies both prior to and immediately (within 15 min) after an intensive 60‐min exercise bout. However, we found no acute regulation of muscle miR‐494 content by intensive exercise (Figure 2b). Interestingly, serum levels of miR‐494 in resting (non‐exercise) state were markedly increased following the 12‐week exercise training programme (*P* = 0.019), and, similar to muscle, were not affected by an acute bout of exercise (Figure 2c). To study the effect of exercise mimicking treatment on the regulation of miR‐494 in vitro, we exposed human differentiated myotubes to EPS using two protocols (Table 3 and Figure 1b). The continuous EPS, stimulation applied continuously for a longer time period, 24–48 h (e.g., 1 Hz, 2 ms, 11.5 V, 24 h), is generally used as a model for exercise training (Nintou et al., 2022). However, this protocol does not reflect the episodic nature of exercise, which is characterised by the alternation of periods with higher intensity exercise and resting periods. Therefore, we developed an intermittent EPS protocol, in which 2‐h subthreshold frequency stimulations (0.2 Hz, 4 ms) were combined with 30‐min high‐frequency stimulation intervals and 5‐min rest periods (Figure 1b). However, we found that neither the continuous nor the intermittent EPS protocol altered miR‐494 levels in cultured human primary skeletal muscle cells or in the cell‐conditioned media (Figure 2d–e). Based on these findings, we conclude that miR‐494 levels in skeletal muscle and circulation are likely not regulated by acute exercise, but rather reflect long‐term adaptive changes associated with exercise training. Moreover, neither continuous nor intermittent (24‐h) EPS of muscle cells recapitulates the effects of 12 months of supervised training on miR‐494 content and release from muscle cells in vitro.

### 3.3 Skeletal muscle miR‐494 levels are not altered in obesity and type 2 diabetes

**FIGURE 3:**
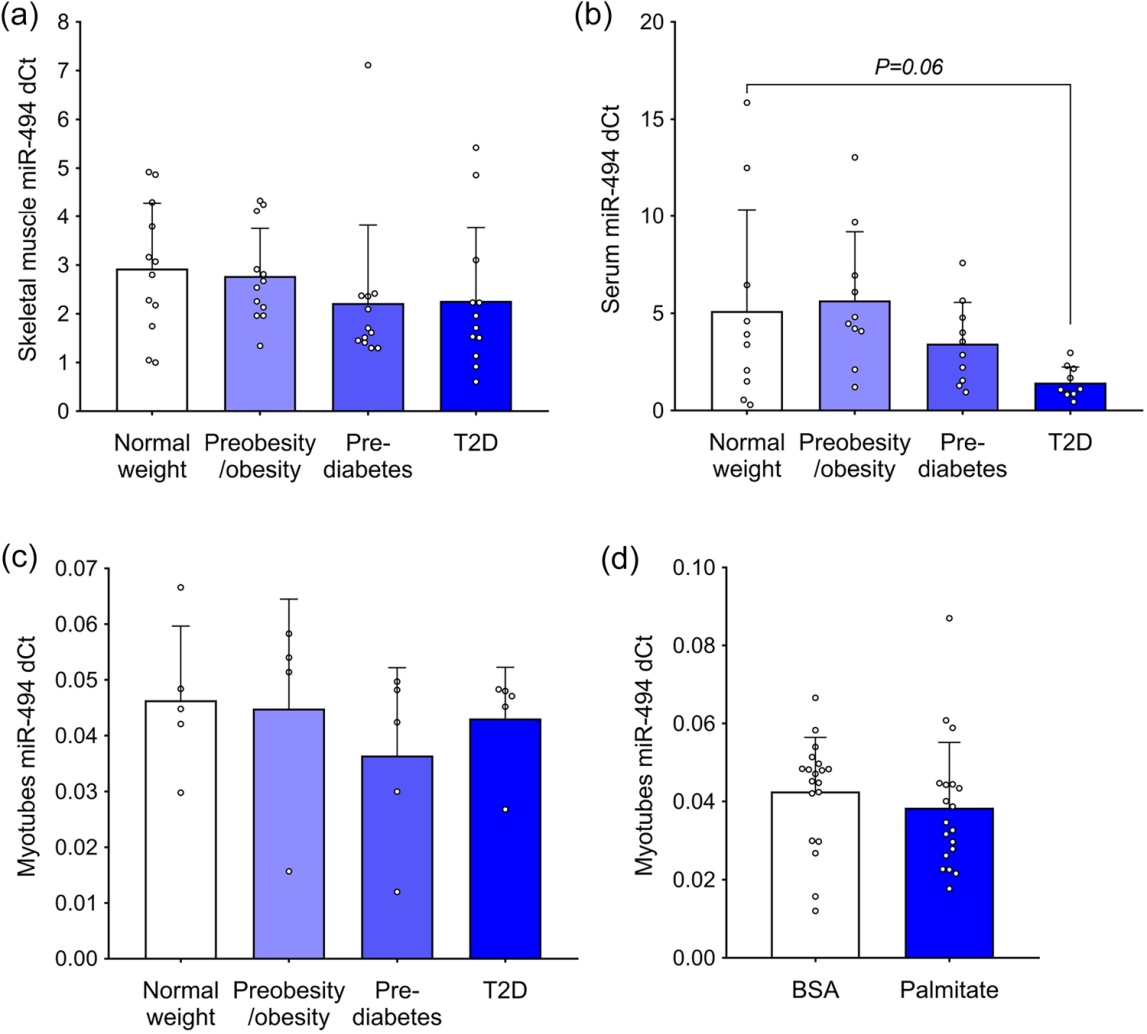
Regulation of skeletal muscle miR‐494 in obesity and metabolic disease. (a) Regulation of miR‐494 in skeletal muscle of lean men (*n* = 12) and patients with obesity (*n* = 12), prediabetes (*n* = 12) and T2D (*n* = 12) at basal state (data are normalised to levels of *RNU6*). (b) Regulation of miR‐494 in serum of lean men (*n* = 10) and patients with obesity (*n* = 10), prediabetes (*n* = 10) and T2D (*n* = 10) at basal state (data are normalised to levels of *RNU6*). (c) Regulation of miR‐494 in skeletal muscle cells of lean men (*n* = 5) and patients with obesity (*n* = 5), prediabetes (*n* = 5) and T2D (*n* = 5) (data are normalised to levels of *RNU6*). (d) Effect of a 3‐day palmitate treatment on miR‐494 levels in differentiated primary human skeletal muscle cells (*n* = 19; data are normalised to levels of *RNU6*).

Next, we studied the regulation of miR‐494 in skeletal muscle from lean control men and patients with pre‐obesity/obesity, prediabetes, or newly diagnosed type 2 diabetes (Cohort 3; Figure 1a). We found that miR‐494 content in the vastus lateralis muscle (Figure 3a) was not significantly altered in men with obesity, prediabetes or type 2 diabetes compared to lean, metabolically healthy men. Similarly, serum miR‐494 levels were not affected in individuals with obesity or prediabetes, but tended to reduce in patients with type 2 diabetes (*P* = 0.06; Figure 3b). We next examined the regulation of miR‐494 by palmitate (an in vitro model of obesity‐associated lipid overload) in primary human skeletal muscle cells in vitro. We showed that muscle cells derived from lean controls and patients with obesity, prediabetes or type 2 diabetes showed no regulation of miR‐494 (Figure 3c). In addition, a 3‐day palmitate treatment did not alter miR‐494 levels in differentiated myotubes (Figure 3d). These findings suggest that skeletal muscle miR‐494 is not regulated by obesity or metabolic disease, nor is it responsive to palmitate treatment in differentiated muscle cells.

### 3.4 MiR‐494 modulates gene expression in human myotubes, in vitro

**FIGURE 4:**
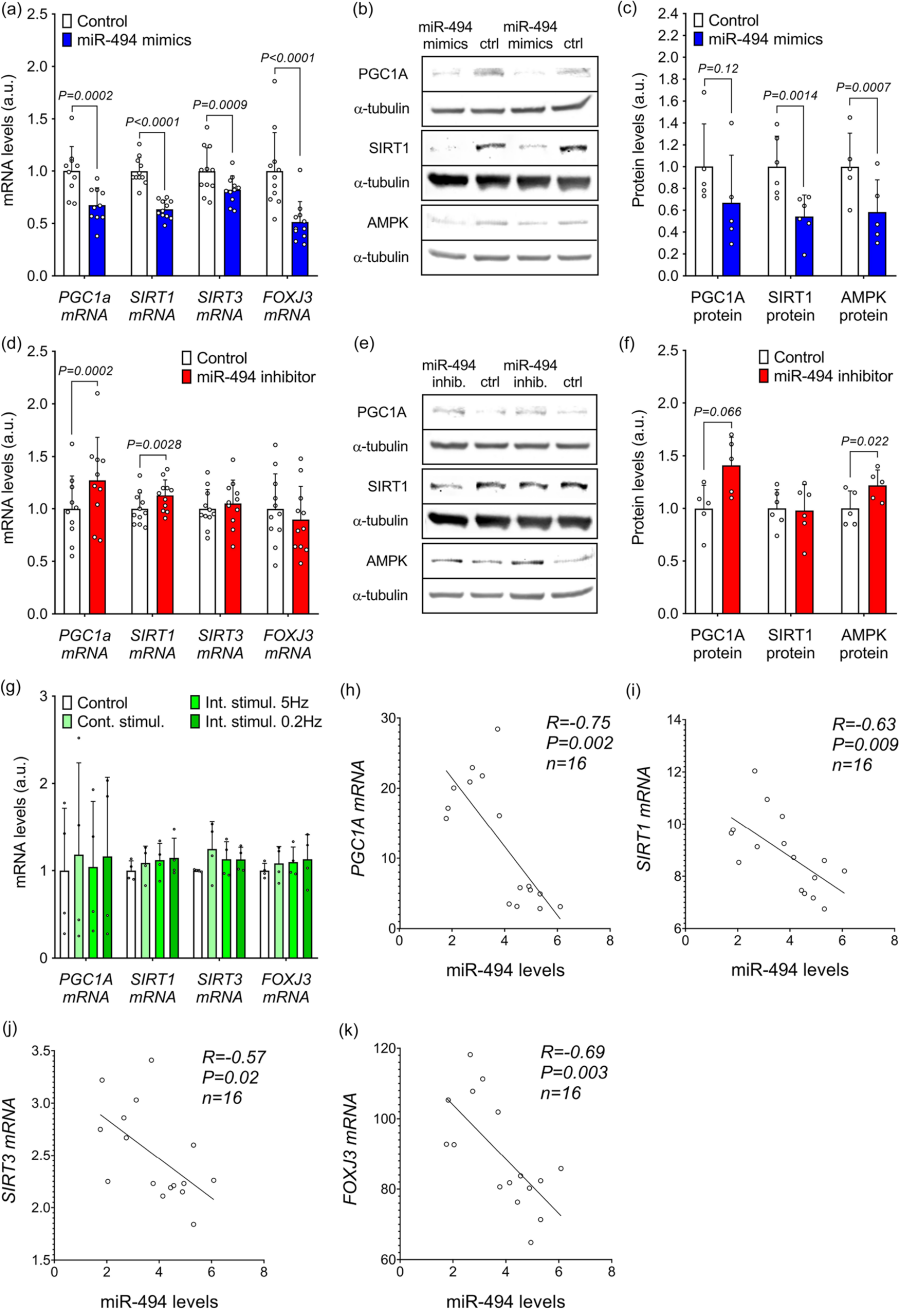
Effect of miR‐494 modulation on mRNA and protein levels of predicted target genes in primary human skeletal muscle cells. (a–c) The effect of miR‐494 mimics transfection on mRNA (a) and protein (b, c) levels of predicted targets (*n* = 10–11 for mRNA, *n* = 5–6 for proteins). (d–f) Effect of miR‐494 inhibitor transfection on mRNA (d) and protein (e, f) levels of predicted targets (*n* = 10–11 for mRNA, *n* = 5–6 for proteins). (g) Effect of EPS on expression of miR‐494 target genes – *PGC1A*, *SIRT1*, *SIRT3* and *FOXJ3*. (h–k) Correlation of miR‐494 with expression levels of *PGC1A* (h), *SIRT1* (i), *SIRT3* (j) and *FOXJ3* (k) in primary human skeletal muscle cells derived from overweight‐to‐obese healthy donors exposed to EPS. Statistical significance was calculated using Student's paired *t*‐test.

Since skeletal muscle miR‐494 is regulated by exercise training and has previously been associated with the regulation of mitochondrial biogenesis, we performed a series of in vitro experiments using primary human skeletal muscle cells differentiated into myotubes to study the role of miR‐494 in the regulation of muscle metabolism. First, we assessed the expression of predicted target genes and proteins involved in lipid oxidation and mitochondrial biogenesis (based on targetscan.org), as well as lipid accumulation and mitochondrial content and function in differentiated primary human myotubes transfected with either a miR‐494 mimic, inhibitor, or the appropriate negative controls. Increase of cellular miR‐494 content achieved by transfection of the miR‐494 mimics significantly lowered the expression of *PGC1A* mRNA (by 33%; *P* = 0.00021), *SIRT1* mRNA (by 37%; *P* = 0.00001), *SIRT3* mRNA (by 18%; *P* = 0.0009) and *FOXJ3* mRNA (by 49%; *P* = 0.00001) (Figure 4a). These transcriptional changes translated into a significant reduction in SIRT1 protein (by 46%; *P* = 0.0014) and AMPK protein (by 42%; *P* = 0.0007), while the levels of PGC1A protein showed a non‐significant trend towards reduction (*P* = 0.12; Figure 4b,c). Conversely, transfection with a miR‐494 inhibitor, which specifically binds and suppresses endogenous miR‐494, increased the expression of *PGC1A* mRNA (by 27%; *P* = 0.00018) and *SIRT1* mRNA (by 17%; *P* = 0.0028), while the expression of *SIRT3* and *FOXJ3* genes was not altered (Figure 4d). Post‐transcriptional changes induced by repression of miR‐494 led to a moderate increase in AMPK protein levels (by 22%; *P* = 0.022) and a trend toward increased PGC1A protein expression (by 41%; *P* = 0.066), while SIRT1 levels were not significantly affected (Figure 4e,f). EPS had no significant effect on the expression of miR‐494 target genes (Figure 4g). However, we found strong negative inter‐relations between miR‐494 levels and the expression of *PGC1A* (*R* = −0.75, *P* = 0.002, *n* = 16), *SIRT1* (*R* = −0.63, *P* = 0.009, *n* = 16), *SIRT3* (*R* = −0.57, *P* = 0.02, *n* = 16) and *FOXJ3* (*R* = −0.69, *P* = 0.003, *n* = 16) in primary human skeletal muscle myotubes exposed to EPS (Figure 4h–k), further supporting the observed regulatory mechanism. Taken together, these data confirm the role of miR‐494 in the control of transcripts encoding key regulators of mitochondrial biogenesis and function in human primary skeletal muscle cells.

### 3.5 MiR‐494 regulates energy metabolism and mitochondrial function in human skeletal muscle cells, in vitro

**FIGURE 5:**
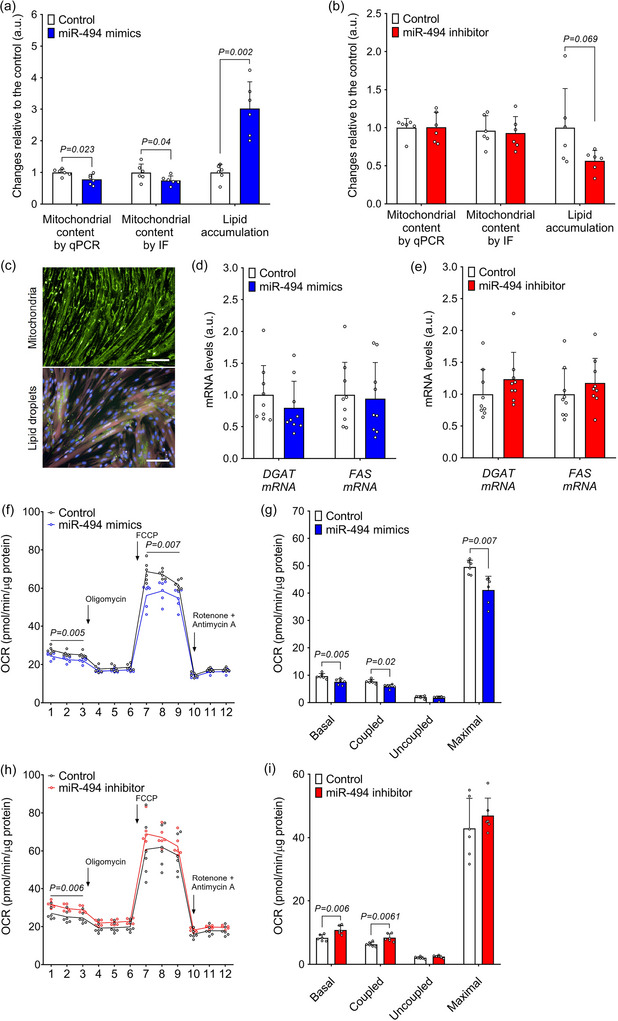
Effect of miR‐494 modulation on mitochondrial function and lipid accumulation in primary human skeletal muscle cells. (a, b) Effects of miR‐494 mimics and inhibitor on mitochondrial content and lipid accumulation (*n* = 6) in primary human skeletal muscle cells. (c) Representative staining of mitochondria using MitoTracker green (green) (upper panel) and lipid droplets, nuclei and cytosol using Bodipy (green), Hoechst (blue) and Syto60 (red) (lower panel). Scale bar = 50 µm. (d–i) Expression of key regulators of lipogenesis *DGAT* and *FAS* mRNA (*n* = 9) (d, e) and mitochondrial respiration (*n* = 6) (f–i) in differentiated primary human skeletal muscle cells. Statistical significance was calculated using Student's *t*‐test. DGAT, diacylglycerol acyltransferase; FAS, fatty acid synthase.

To find out whether the effects of miR‐494 on *PGC1A*, *SIRT1* and *AMPK* mRNA and protein levels translate into functional changes, we focused on quantification of lipid accumulation by immunofluorescence. Transfection with miR‐494 mimics led to a marked increase (3‐fold; *P* = 0.002), while its inhibition tended to reduce lipid accumulation (by 44%; *P* = 0.069) in differentiated primary human myotubes (Figure 5a–c). To find out whether miR‐494 affects lipogenesis, we quantified the expression of key lipogenic enzymes, including *DGAT* and *FAS*. However, the expression of neither enzyme was significantly altered by miR‐494 manipulation (Figure 5d,e). Interestingly, transfection with miR‐494 mimics resulted in a significant reduction in mitochondrial content (by 22%; *P* = 0.023), while its inhibition did not alter the number of mitochondria (Figure 5a–c) in primary human skeletal muscle cells. This effect was confirmed using two independent methods: MitoTracker green staining assessed by immunofluorescence (24.8% reduction in response to miR‐494 mimics, *P* = 0.040) and qPCR‐based quantification of mitochondrial DNA content (22% reduction in response to miR‐494 mimics, *P* = 0.023). We also tested the effect of miR‐494 manipulation on mitochondrial respiration by measuring oxygen consumption and found a significant reduction of basal (*P* = 0.005), coupled (*P* = 0.002) and maximal (*P* = 0.007) mitochondrial respiration in response to miR‐494 mimics (Figure 5f,g). Conversely, inhibition of miR‐494 enhanced both basal (*P* = 0.006) and coupled (*P* = 0.0061) mitochondrial respiration (Figure 5h,i). These findings clearly suggest that miR‐494 acts as a negative regulator of both mitochondrial biogenesis and activity in human skeletal muscle cells.

### 3.6 MiR‐494 inhibition increases *Pgc1a* mRNA levels in murine tibialis anterior muscle, in vivo

**FIGURE 6:**
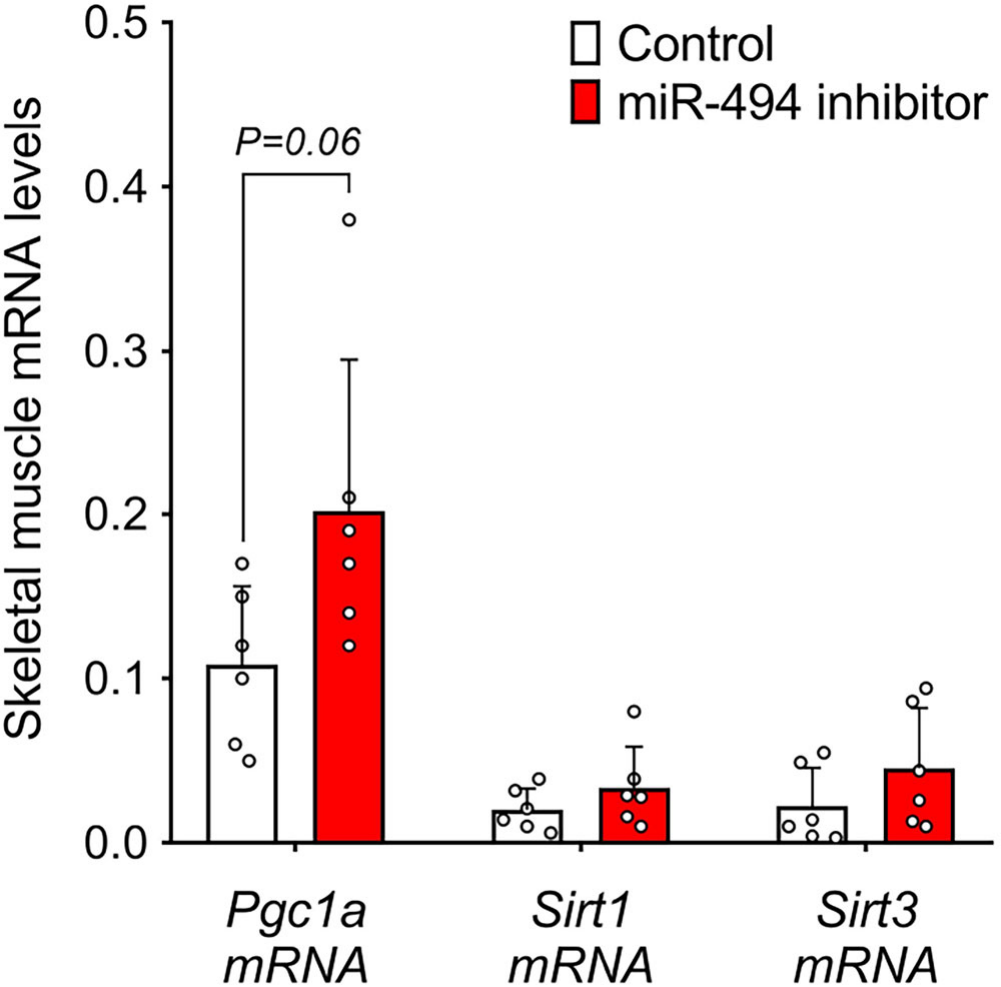
Effect of miR‐494 inhibition on *Pgc1a*, *Sirt1* and *Sirt3* expression in tibialis anterior muscle of mice (*n* = 6). Statistical significance was calculated using Student's paired *t*‐test.

To test the efficacy of miR‐494 inhibition in vivo, we injected a miR‐494 inhibitor and the respective negative control into the tibialis anterior muscles of 12‐week‐old C57Bl/6 mice. In this proof‐of‐concept experiment, inhibition of miR‐494 tended to increase *Pgc1a* mRNA levels (by 87%; *P* = 0.06), while the expression levels of *Sirt1* and *Sirt3* were not changed (Figure 6a). Based on these data, we conclude that miR‐494 might play an important role in the regulation of mitochondrial function in skeletal muscle, potentially through modulation of PGC1A. However, further studies are required to validate its functional significance in muscle physiology and whole‐body energy homeostasis.

## 4 DISCUSSION

MiRNAs play an important role in the regulation of cellular function by fine‐tuning the expression of their target genes. Several miRNAs have been shown to control mitochondrial function and energy metabolism in skeletal muscle. Here, we provide evidence that skeletal muscle miR‐494 is regulated by exercise training but is not affected by acute exercise or the presence of obesity and metabolic disease. In addition, we demonstrate that genetic manipulation of miR‐494 affects mitochondrial biogenesis and respiration, as well as lipid metabolism, in cultured primary human skeletal muscle cells through modulation of PGC1A, SIRT1 and AMPK levels. Moreover, in a proof‐of‐concept experiment, we show that inhibition of miR‐494 tends to increase skeletal muscle *Pgc1a* mRNA levels in vivo in mice, further validating the observed regulatory mechanism and supporting the role of miR‐494 as an important regulator of mitochondrial biogenesis and function.

It is known that the type, intensity and duration of an acute exercise bout can modulate both muscle and circulating miRNAs (Siracusa et al., 2018). Based on our data, it appears that skeletal muscle miR‐494 levels are not regulated by acute exercise in either sedentary or trained individuals, but rather respond to long‐term adaptive changes in active muscle. Previously, Yamamoto et al. (2012) reported that miR‐494 content in the gastrocnemius muscle of C57BL/6J mice significantly decreased after 7 days of swimming exercise (105 min per day). However, it remains unclear whether the reduction in miR‐494 levels measured in muscle 2 h after exercise was due to the acute exercise bout or the preceding 7 days of training (Yamamoto et al., 2012). Similarly, 8 weeks of voluntary wheel running led to a significant reduction of miR‐494 in the gastrocnemius muscle of C57BL/6J mice (Sun et al., 2016). In contrast, 4‐week exercise programmes did not result in a significant reduction in miR‐494 expression in wild‐type mice. Notably, research on CBS± mice, which exhibit greater fatigue and reduced contractile force, revealed elevated levels of miR‐494 in skeletal muscle, with a significant decrease in expression following a 4‐week aerobic endurance training programme (Veeranki et al., 2015). In agreement with our findings and previously published observations, EPS had no significant effect on miR‐494 levels in muscle cells in vitro. The expression of miR‐494 has been shown to be suppressed during myogenic induction in C2C12 myoblasts (Yamamoto et al., 2012). Interestingly, we found an increase in circulating miR‐494 levels in trained volunteers, with no effect seen following acute physical exercise. Notably, previous studies have shown that miR‐494 is upregulated under muscle atrophy conditions (Greco et al., 2009) and downregulated during regeneration and hypertrophy (Iwasaki et al., 2015; Sun et al., 2016). These findings suggest that enhanced myogenesis, reflecting an increase in training‐induced muscle regeneration capacity, could be one potential mechanism underlying miR‐494 downregulation in trained muscle. Elevated basal levels of circulating miR‐494 have also been detected in athletes compared to healthy controls (Denham & Prestes, 2016). The reciprocal regulation of miR‐494 in muscle and serum observed in our study suggests that miR‐494 may be released from muscle into the circulation. Thus, trained muscle could serve as a source of circulating miR‐494, and this release may at least partially explain the relative downregulation of miR‐494 in the muscle of trained individuals. However, since miR‐494 is expressed in multiple tissues, further studies are needed to elucidate the origin, fate and function of training‐induced miR‐494 in the bloodstream (Sapp et al., 2017; Silva et al., 2017). Increasing evidence suggests that circulating miRNAs may play a role in exercise‐induced tissue crosstalk, which is important for the systemic adaptive response to exercise.

Although gestational diabetes mellitus has been associated with downregulation of circulating miR‐494 in He et al. (2017), our findings show no difference in muscle miR‐494 content among sedentary men when comparing lean controls with individuals with pre‐obesity/obesity, prediabetes and type 2 diabetes. Furthermore, a 3‐day palmitate treatment of cultured, differentiated myotubes had no effect on miR‐494 levels, suggesting that skeletal muscle miR‐494 is not altered under obesogenic conditions that promote the progression of metabolic disease.

Our study revealed that miR‐494 targets key regulators of mitochondrial function, including *PGC1A*, *SIRT1*, *SIRT3* and *FOXJ3*. The regulation of mitochondrial biogenesis by miR‐494 has previously been shown to be mediated through TFAM and FOXJ3 (Yamamoto et al., 2012), and associated with PGC1A, which was significantly upregulated following 8 weeks of voluntary wheel running in mice. Here, we provide the first evidence of the role of miR‐494 in regulating mitochondrial biogenesis in a human model that preserves the metabolic phenotype of the cell's donor (Ukropcova et al., 2005). It is well known that regular exercise promotes mitochondrial biogenesis as a result of gene expression changes induced by muscle contraction. Exercise activates several adaptive mechanisms in skeletal muscle, including the activation of AMPK, the cell's metabolic and energy sensor, which enhances glycolysis (Hardie, 2007; Ojuka et al., 2000), fat oxidation (Civitarese et al., 2006) and the expression of genes involved in mitochondrial respiration (Winder et al., 2000) and lipid breakdown (Zhou et al., 2001). It has been documented that AMPK is activated in skeletal muscle during exercise and following EPS‐induced contraction of cultured skeletal muscle cells (Vavvas et al., 1997; Winder & Hardie, 1996). In addition, EPS induces mitophagy in C2C12 myotubes by activating the AMPK‐ULK1 pathway (Gao et al., 2020). AMPK activation leads to increased expression of *PGC1A* (Suwa et al., 2003; Terada et al., 2002) and regulates genes involved in mitochondrial and glucose metabolism (Jäger et al., 2007). SIRT1, another regulator of PGC1A, has been associated with enhanced mitochondrial activity, improved exercise performance and increased thermogenic activity (Lagouge et al., 2006). SIRT1 is also influenced by AMPK activation, which can increase cellular levels of NAD^+^, a cofactor required for SIRT1 activity (Lin et al., 2004). In turn, SIRT1 forms a feedback loop that helps optimise the cellular response to energy fluctuations by activating proteins that enhance AMPK signalling (Ruderman et al., 2010). Inhibition of miR‐494 led to a significant increase in *PGC1a* and *SIRT1* mRNA, as well as AMPK protein levels. Therefore, it is reasonable to assume that the downregulation of miR‐494 contributes to the induction of mitochondrial biogenesis in trained muscle. However, in addition to miR‐494, several other miRNAs have also been shown to regulate muscle metabolism by targeting *PGC1A*, including miR‐29 (in the cardiovascular system) (Caravia et al., 2018), miR‐761 (in C2C12 myotubes) (Xu et al., 2015), miR‐696 (in C2C12 myotubes and SNARK‐transgenic mice) (Queiroz et al., 2021), miR‐23 (in the quadriceps femoris muscle C57BI/6J mice) (Safdar et al., 2009) and miR‐204 (in C2C12 myotubes) (Houzelle et al., 2020). Our study is the first to demonstrate that modulation of miR‐494 affects lipid accumulation, as well as mitochondrial content and respiration, in primary human skeletal muscle cells. The inhibitory effect of miR‐494 on mitochondrial OCR was previously reported by Iwasaki et al. (2021, 2015) in human induced pluripotent stem (hiPS) cells. The stronger effects observed with miR‐494 mimics, compared to the inhibitor, on target gene expression, mitochondrial content and lipid accumulation may be due to compensatory mechanisms involving other miRNAs or regulatory pathways that offset the loss of miR‐494. We also sought to validate the observed regulatory mechanism in vivo through a proof‐of‐concept experiment utilising the injection of a miR‐494 inhibitor into the tibialis anterior muscle of mice. This intervention showed a trend towards increased *Pgc1a* mRNA levels (*P* = 0.06). However, further studies are required to confirm the role of miR‐494 in regulating mitochondrial function and lipid metabolism in skeletal muscle in vivo, as well as to assess the therapeutic potential of miR‐494 inhibition.

### 4.1 Study limitations

Prediabetes was diagnosed in our study based on the results of an oral glucose tolerance test, but the levels of HbA1C are not available. Our study provides evidence for a specific role of miR‐494 in the regulation of mitochondrial function and lipid metabolism, using both cross‐sectional and interventional clinical studies, as well as a unique cell culture model of primary human muscle cells differentiated into myotubes. This model retains the metabolic phenotypes of the donors, and therefore more closely resembles in vivo muscle physiology. However, it also introduces high interindividual variability, which may limit our ability to detect the effects of EPS, a method that itself is subject to considerable individual variation. Additionally, the use of serum for quantifying circulating miR‐494 levels may introduce variability and potential contamination, as coagulation can trigger the release of cellular components, including miRNAs, from platelets and other blood cells. Another limitation of our study is that it focused on a selected set of target genes and assessed only specific aspects of mitochondrial function, without a more comprehensive investigation of downstream functional outcomes. The proof‐of‐concept mouse experiment was designed to mimic the exercise training‐induced decline in muscle miR‐494 levels. The miR‐494 inhibitor was injected directly into the tibialis anterior muscle of mice. We expected that injection of the inhibitor would induce local suppression of miR‐494 levels along the tibialis anterior muscle. This experiment provided an opportunity to validate miR‐494 target genes and investigate the regulatory mechanisms associated with the reduction of miR‐494 in vivo. However, due to its localised nature, the experiment did not assess systemic regulation of miR‐494 or the effects of inhibitor injection on whole‐body energy metabolism. Future studies are needed to evaluate the impact of miR‐494 inhibition on skeletal muscle function and systemic energy homeostasis. A deeper understanding of the mechanisms by which miR‐494 operates could offer valuable insights into the potential therapeutic application of its inhibition. Despite these limitations, our study makes a significant contribution to the understanding of the role of miR‐494 in the adaptive response to exercise and highlights miR‐494 as a promising target for the treatment of obesity and associated metabolic disorders.

## AUTHOR CONTRIBUTIONS

All authors contributed to the study conception and design. Experimental work, data collection and analysis were performed by Miroslav Baláž, Natália Pálešová, Klára Gabrišová, Jana Babulicová, Patrik Krumpolec, Zuzana Kovaničová, Tímea Kurdiová and Salvatore Modica. Clinical studies were performed by Barbara Ukropcová, Jozef Ukropec, Tímea Kurdiová and Miroslav Baláž. Funding was acquired by Barbara Ukropcová, Jozef Ukropec, Miroslav Baláž and Christian Wolfrum. The study was designed by Barbara Ukropcová, Jozef Ukropec, Christian Wolfrum and Miroslav Baláž. The first draft of the manuscript was written by Miroslav Baláž, Natália Pálešová, Klára Gabrišová, and all authors commented on previous versions of the manuscript. All authors have read and approved the final version of this manuscript and agree to be accountable for all aspects of the work in ensuring that questions related to the accuracy or integrity of any part of the work are appropriately investigated and resolved. All persons designated as authors qualify for authorship, and all those who qualify for authorship are listed.

## CONFLICT OF INTEREST

The authors declare no conflicts of interest.

## Data Availability

All data supporting the results are presented in the paper and are available upon request.
